# Towards a ‘wide’ role for venture capital in OECD countries' industry 4.0

**DOI:** 10.1016/j.heliyon.2021.e08700

**Published:** 2022-01-03

**Authors:** Bruno S. Sergi, Elena G. Popkova

**Affiliations:** a Harvard University, USA; b University of Messina, Italy; c Moscow State Institute of International Relations (MGIMO University), Moscow, Russian Federation

**Keywords:** Venture capital, Industry 4.0, Competitiveness, Digital technologies

## Abstract

This paper focuses on the current theoretical views of venture capital that predetermines a "narrow" treatment. In the light of the existing "narrow" treatment, venture investors seek private commercial interests in financial support for Industry 4.0, ignoring other interests that fall beyond the limits of the current "narrow" treatment of venture capital. A "wide" treatment of venture capital 4.0 proposed in this paper allows for improving venture investors' market strategies. Implementing this treatment, they will strive for providing a whole range of advantages for society. Due to this novel approach, venture capital 4.0 might become a tool of corporate social responsibility. To substantiate this novel approach, this paper considers data for 2020 that reflect the influence of venture capital 4.0 on the economy in the period of its stability for 33 countries of the OECD, including developed and developing countries. Econometric modelling based on the official statistics data proves that Industry 4.0 venture capital will help achieve such growth goals as innovative development, global competitiveness, and increasing digital competitiveness. The limitations of this research are due to the impossibility of achieving such goals as sustainable development, economic growth, and implementation of human potential; what's more, the specifics of developing countries have not been studied sufficiently. The conclusions are oriented mainly at developed countries and could merely partially be applied to developing countries. During further research, it is expedient to explore – more thoroughly – the experience of the influence of Industry 4.0 venture capital on emerging economies.

## 1 Introduction

The global economy is boosting the transition to a new technological mode of Industry 4.0 (e.g., Park and Kim, 2018; Popkova et al., 2013, 2018; Popkova and Sukhodolov, 2017). The features of Industry 4.0 are a significant increase in efficiency, the population's living standards, and added possibilities for strengthening human potential. The uniqueness of the Fourth Industrial Revolution is due to multiple reasons. First, the digital technologies of Industry 4.0 systemically cover all spheres of economic activities. The first three industrial revolutions changed only the technologies of industrial production. In contrast, the Fourth Industrial Revolution shaped everything, ranging from the creation of fully autonomous "smart" (robotised) companies to remote employment and online communications (Lee and Jung, 2018; Prinz et al., 2018).

Second, the Fourth Industrial Revolution extends to all business processes, including production and management, marketing, HR management, distribution, sales, and consumption. Transformations that are caused by the transition to Industry 4.0 take place not only in industry but also in agriculture (“smart” farms) and service sphere (online commerce), including public services that develop based on e-government (Bogoviz et al., 2018; Niemann and Pisla, 2018).

Third, the Fourth Industrial Revolution relies not only on technical (technological progress, like all previous industrial revolutions) but also socially, which is equal and maybe even more significant. The knowledge society, strung with digital information and supporting knowledge-intensive employment in the sphere of high technologies, is a new social phenomenon that lies based on Industry 4.0. According to international experience, the most active participants of the Fourth Industrial Revolution are countries with the most progressive societies; therefore, Industry 4.0 exceeds the limits of the economy; it is essential to study its implications for society.

The moving force of the industrial revolution is venture capital. It redirects the investment flows to Industry 4.0. The nature of the Fourth Industrial Revolution allows supposing that it assigns a new value to venture capital and the current theoretical views of venture capital led to its "narrow" treatment, limited by its strictly fixed advantages for the economy.

Guided by the existing “narrow” treatment, venture investors seek mainly private commercial interests in financial support for Industry 4.0, ignoring other interests of concerned parties, which are poorly studied and not determined by economics (since they are beyond the limits of the existing “narrow” treatment of venture capital).

While national governments are embarking on digital modernization strategies and actively financing R&D of breakthrough digital technologies, private entrepreneurship barely assesses the involvement in this process (Bonaventura et al., 2020; DeFrancesco, 2021; Della Giovampaola and Engheta, 2014; Li et al., 2020; Rodríguez et al., 2021). This does not allow developing the potential of venture capital in the sphere of the generation of advantages for economic systems. Subsequently, this paper explores a new role for venture capital in Industry 4.0. This paper's novelty is the broad goal of checking the Hypothesis that industry 4.0 venture capital would help achieve all purposes of this growth: sustainable and innovative development, global competitiveness, implementation of human potential, and increasing its digital competitiveness. Technological progress, investment, and innovation theories support this paper's research question. This introduction is followed by the literature review, methodology, results, discussion, and conclusions.

## 2 Literature review

### 2.1 The concept and technologies of industry 4.0

Industry 4.0 is expected to be fertile for many areas. Such authors as Ayinde and Kirkwood (2020), Amri et al. (2019), Kummitha (2019), Loureiro (2018), Popkova et al. (2019), Ragulina et al. (2019), Popkova et al. (2018), Popkova and Sergi (2018), Vecchio et al. (2019), Yu et al. (2020) apply the terms “the Fourth industrial revolution”, “digital modernisation of the economy”, and “neo-industrialisation”. The breakthrough 4.0 technologies are as follows:

– Internet of Things (IoT), studied by Ansari et al. (2018), Mendoza and Kleinschmidt et al. (2018);
– Blockchain (Li et al., 2018; Ma et al., 2018);
– The technology of processing Big Data allows for automatisation of processing vast arrays of digital information (e.g., Kumar et al., 2018; Kumari et al. (2018);
– Unmanned flying vehicles and manipulators – remotely controlled mechanisms – are investigated by Dehghani and Menhaj (2018) and Song et al. (2018);
– Robototronics (Jaikaew et al., 2018; Shafei and Shafei, 2018);
– Virtual and alternate (Abiri et al., 2018; de-Juan-Ripoll et al., 2018);
– The technology of 3D print (Abiri et al., 2018; de-Juan-Ripoll et al., 2018; Dickinson, 2018).
– Artificial intelligence (AI) (Galloway and Swiatek, 2018).

Based on national strategies, these technologies are tested worldwide. “Advanced Manufacturing” and “Initiatives for Global Competitiveness” in the US; in Germany – “High-Tech Strategy Innovations”, in France – “La Nouvelle Industrielle”, in the UK – “Eight Great Technologies”, and in Russia – breakthrough (leading) digital technologies”. The gaps in financially supporting the digital transition to Industry 4.0 are poorly investigated and on the whole the financing of Industry 4.0 has been studied insufficiently.

### 2.2 Advantages of industry 4.0 for sustainable development

Transition to Industry 4.0 is not a goal per se, but a tool for achieving global goals of modern humankind; this includes:

- Supporting the implementation of the SDGs (Aldieri and Vinci, 2018; Marques et al., 2018);
- Formation of competitive advantages (Krammer, 2017; Kuhlman et al., 2017);
- Accelerated growth of GDP (Bijlsma et al., 2018; Wang et al., 2018);
- Development of labour potential and the most effective use of human capital (Ayinde and Kirkwood, 2020; Laybats and Tredinnick, 2020; Yessengeldin et al., 2015).
- Development of the knowledge society and knowledge economy (innovative economy) (Wei and Yu, 2018);
- Strengthening the digital competitive advantages and positions in the high-tech world markets (Teece, 2018; Trappey et al., 2016).

Systemic achievement of the above goals ensures economic and public well-being growth. A shortcoming of the current works is a lack of elaboration on Industry 4.0 from economic efficiency. Existing publications focus on the overlooked costs or indirectly considered costs, the value of which is equally important.

Sustainable development has been examined in Guo et al. (2021), Jost et al. (2021), Lindsay et al. (2021), Miklosik and Evans (2021), Mondejar et al. (2021), Timpabi et al. (2021), and Wang and Huang (2021). The contribution of Industry 4.0 to sustainable development has been noted by Harikannan et al. (2021), Mhlanga (2021), Khan et al. (2021), and Gupta et al. (2021). The necessity to use the technologies of Industry 4.0 for sustainable development is explained, first, by the growing need for investments and consumer goods, which is satisfied with the help of venture capital in Industry 4.0 and, second, by the necessity to ensure a balance between environment, economy, and society, which is stimulated by the Sustainable Development Goals, which are integrated into Industry 4.0.

### 2.3 The current “narrow” economic treatment of venture capital

According to Aggarwal and Elembilassery (2018), Breuer and Pinkwar (2018), Ceccagnoli et al. (2018), and Bock et al. (2018), Chung and Kang (2018), Dziallas (2020), Friske and Zachary (2019), Hunt et al. (2019), Jiang and Zhao (2019), Sindakis et al. (2019), Wei et al. (2018), Xue et al. (2020), Zhang (2019), Zubair et al. (2020), venture capital has the following specific features:

– Support for breakthrough (extraordinary) technologies;
– High risk;
– Strategic (without short-term or mid-term return) investments.

According to Amona et al. (2018), Anggusti and Siallagan (2018), Breznitz et al. (2018), Cumming and Schwienbacher (2018), Guerini and Tenca (2018), Kelly and Kim (2018), Sargon and Katircioğlu (2018), Pogodina et al. (2018) and Wen et al. (2018), venture capital is actively used. The content analysis of the above literature has shown that it does not sufficiently define the contribution of venture capital of Industry 4.0 to sustainable development. Our position as to the results of the literature analysis is as follows: incomplete consideration of consequences of the use of venture capital in Industry 4.0 contradicts the concept of sustainable development and hinders its practical implementation. Considering the necessity to ensure a balance between environment, economy, and society for integrating the Sustainable Development Goals in Industry 4.0, this paper explores the consequences of venture capital.

## 3 Methodology

The research methodology is based on the regression analysis and our research Hypothesis is as follows.

Hypothesis Industry 4.0 venture capital will help achieve all goals of this growth: sustainable and innovative development, global competitiveness, implementation of human potential, and increasing its digital competitiveness (Bonilla al., 2018; Ghobakhloo, 2020; Oláh et al., 2020). For better visualization of the data, we adopt the following legend:

– x = Venture capital investments, percentage of GDP (according to the OECD);
– y_1 =_ Global Sustainable Development Index (according to the Sustainable Society Foundation), points 1–100;
– y_2 =_ Global Competitiveness Index (according to the World Economic Forum), points 1–7;
– y_3 =_ Annual growth rate of GDP in constant prices (according to the International Monetary Fund);
– z_1 =_ Human Development Index (according to the UNDP), points 0–1;
– z_2 =_ Global Innovation Index (according to the WIPO), points 1–100;
– z_3 =_ Digital Competitiveness index (according to the IMD), points 1–100.

We analyze 33 countries for which official statistical information (2020) is available. The group of countries, which is the sample for this research, is sufficient for the correct reflection of the influence of Industry 4.0 venture capital on the economy since it includes developed and developing countries and covers all geographic regions of the world (parts of the world). However, it should be acknowledged that developed countries dominate in the sample of the OECD countries. This research reflects the leading experience of using Industry 4.0 venture capital in the modern economy primarily. The experience of developing countries has to be further studied in detail.

## 4 Results

### 4.1 Verification of Hypothesis

Since a global economic crisis took place in 2020, to avoid a distortion of the results of econometric modelling, more reliable data are used in this paper – the data for 2019, which reflect the influence of venture capital of Industry 4.0 on the economy in the period of its stability. The annual growth rate is calculated between 2019 and 2020 (Table 1).

**Table 1:** Statistical data for the selected countries for 2020.

Country	Global Sustainable Development Index	Global Competitiveness Index 4.0	Annual GDP growth rate in constant prices	Human Development Index	Global Innovation Index	Digital Competitiveness index	Venture capital investments, percentage of GDP
	y1	y2	y3	z1	z2	z3	x1
Australia	73.9	78.7	2.945	0.938	50.34	88.897	0.034
Austria	81.1	76.6	1.254	0.914	50.94	84.473	0.020
Belgium	78.9	76.4	1.477	0.919	50.18	82.491	0.078
Germany	81.1	81.8	1.415	0.939	58.19	86.216	0.055
Denmark	85.2	81.2	1.801	0.930	58.44	95.225	0.098
Israel	71.5	76.7	2.964	0.906	57.43	86.373	-
Ireland	78.2	75.1	3.014	0.942	56.10	85.863	0.054
Spain	77.8	75.3	2.045	0.893	47.85	78.743	0.044
Italy	75.8	71.5	0.800	0.883	46.30	67.903	0.014
Canada	77.9	79.6	1.843	0.922	53.88	90.836	0.192
Latvia	77.1	67.0	3.583	0.854	43.23	72.437	0.012
Luxembourg	74.8	77.0	3.308	0.909	53.47	84.368	0.024
Netherlands	80.4	82.4	1.732	0.933	61.44	94.261	0.064
Norway	80.7	78.1	2.098	0.954	51.87	93.671	0.038
Poland	75.9	68.9	2.992	0.872	41.31	73.707	0.019
Portugal	76.4	70.4	1.160	0.850	44.65	73.007	0.018
Republic of Korea (South Korea)	78.3	79.6	3.027	0.906	56.55	91.297	0.164
Russia	70.9	66.7	1.500	0.824	37.62	70.406	0.008
Slovakia	76.2	66.8	3.900	0.857	42.05	62.624	0.017
UK	79.4	81.2	1.606	0.920	61.30	88.691	0.103
USA	74.5	83.7	2.121	0.920	61.73	100.000	0.633
Finland	82.8	80.2	1.511	0.925	59.83	93.732	0.120
France	81.5	78.8	1.749	0.891	54.25	82.522	0.085
Czech Republic	80.7	70.9	2.263	0.891	49.43	71.812	0.011
Switzerland	78.8	82.3	1.600	0.946	67.24	94.648	0.084
Sweden	85.0	81.2	2.181	0.937	63.65	96.070	0.076
Estonia	80.2	70.9	2.708	0.882	49.97	78.669	0.126
Japan	78.9	82.3	0.846	0.915	54.68	82.775	-
Hungary	76.9	65.1	2.600	0.845	44.51	65.472	0.083
New Zealand	79.5	76.7	2.563	0.921	49.55	86.026	0.036
South Africa	61.5	62.4	2.198	0.705	34.04	60.865	-

We performed a series of equations y_1_-y_6_ (Tables 3, 4, 5, 6, 7, and 8). The formal model is as follows:

y_1_ = 78.2033 + 3.0666x
y_2_ = 73.9162 + 21.8888x
y_3_ = -4.6010 + 2.3010x
z_1_ = 0.9005 + 0.0924x
z_2_ = 47.7748 + 30.7468x
z_3_ = 73.1634 + 61.3670x

To ensure the reliability of econometric models, they are compiled according to the Gauss-Markov theorem. For this, we carried a test for multicollinearity of dependent variables out (Table 2).

**Table 2:** Results of test for multicollinearity of dependent variables.

Correlation	y_1_	y_2_	y_3_	z_1_	z_2_	z_3_
y1	1	-	-	-	-	-
y2	0.56	1	-	-	-	-
y3	-0.22	-0.34	1	-	-	-
z1	0.74	0.83	-0.13	1	-	-
z2	0.60	0.91	-0.21	0.80	1	-
z3	0.53	0.92	-0.17	0.81	0.88	1

The test results in Table 2 showed no overlapping variables since none of the correlation coefficients exceeded 0.95. Therefore, there is no multicollinearity in the dependent variables. A test for heteroscedasticity was also carried out - an unequal (non-constant) variance of the random error of regression models, which revealed no heteroscedasticity. This allows the calculation and analysis of standard errors in White's form.

According to the data of Table 3, venture capital does not have a statistically significant influence on the sustainability of development (regression model y_1_ = F(x), Table 2), as significance F = 0.6268 (exceeds 0.05) and change of dependent variable is by 0.0080% explained by the change of independent variable (R^2^ = 0.0080), connection indicators is direct (estimate coefficient is 3.0666). F_obs_ equals 0.2414. F_tabl_ with 32 observations (k_1_ = 32) and 1 variable (k_2_ = 32-1-1 = 31), according to Fisher's F-distribution table, equals 1.89. Since F_obs_ < F_tabl_ (0.2414 < 1.89), the F-test has not been passed at the significance level of 0.05).

**Table 3:** Results of regression analysis of dependence y_1_(x).

Regression statistics	
Multiple R	0.0893
R-square	0.0080
Adjusted R-square	-0.0251
Standard error	3.9453
Observations	32

Dispersion analysis					
	df	SS	MS	F	Significance F
Regression	1	3.7574	3.7574	0.2414	0.6268
Residue	30	466.9692	15.5656		
Total	31	470.7266			

	Coefficients	Standard error	t-statistics	R-Value	Lower 95%	Upper 95%
Y-intercept	78.2033	0.8303	94.1844	0.0000	76.5076	79.8991
x1	3.0666	6.2416	0.4913	0.6268	-9.6804	15.8136

According to the data of Table 4, venture capital has a statistically significant influence on the global competitiveness (regression model y_2_ = F(x), Table 3), as significance F = 0.0174 (does not exceed 0.05) – however, this influence is minimal, as the change of dependent variable is by 13.42% explained by the change of independent variable (R^2^ = 0.1744), connection indicators is direct (estimate coefficient is 21.8888). Fobs equals 6.3374. F_tabl_, with 32 observations (_k1=_32) and 1 variable (k_2_ = 32-1-1 = 31), according to Fisher's F-distribution table, equals 1.89. Since Fobs > Ftabl (6.3377 > 1.89), the F-test has been successfully passed at the significance level of 0.05.

**Table 4:** Results of regression analysis of dependence y_2_(x).

Regression statistics	
Multiple R	0.4176
R-square	0.1744
Adjusted R-square	0.1469
Standard error	5.4960
Observations	32

Dispersion analysis					
	df	SS	MS	F	Significance F
Regression	1	191.4364	191.4364	6.3377	0.0174
Residue	30	906.1856	30.2062		
Total	31	1,097.6220			

	Coefficients	Standard error	t-statistics	R-Value	Lower 95%	Upper 95%
Y-intercept	73.9162	1.1567	63.9041	0.0000	71.5539	76.2784
x1	21.8888	8.6948	2.5175	0.0174	4.1317	39.6459

According to the data of Table 5, venture capital does not have a statistically significant influence on the growth rate (regression model y_3_ = F(x), Table 4), as significance F = 0.6146 (exceeds 0.05), the change of dependent variable is by 0.86% explained by the change of independent variable (R^2^ = 0.0086). However, the connection between the indicators is direct (2.3010). Fobs equals 0.26. F_tabl_, with 32 observations (k_1_ = 32) and 1 variable (k_2_ = 32-1-1 = 31), according to Fisher's F-distribution table, equals 1.89. Since F_obs_ < F_tabl_ (0.26 < 1.89), the F-test has not been passed at the significance level of 0.05.

**Table 5:** Results of regression analysis of dependence y_3_(x).

Regression statistics	
Multiple R	0.09
R-square	0.01
Adjusted R-square	-0.02
Standard error	2.86
Observations	32

Dispersion analysis					
	df	SS	MS	F	Significance F
Regression	1	2.12	2.12	0.26	0.61
Residue	30	245.06	8.17		
Total	31	247.17			

	Coefficients	Standard error	t-statistics	P-Value	Lower 95%	Upper 95%
Y-intercept	-4.60	0.60	-7.65	0.00	-5.83	-3.37
x1	2.30	4.52	0.51	0.61	-6.93	11.54

According to the data of Table 6, venture capital does not have a statistically significant influence on human development (regression model y_4_ = F(x), Table 5), as significance F = 0.2387 (exceeds 0.05), the change of dependent variable is by 4.60% explained by the change of independent variable (R^2^ = 0.0460). However, the connection between the indicators is direct (estimate coefficient = 0.0924). Fobs equals 1.4452. F_tabl_, with 32 observations (k_1_ = 32) and 1 variable (k_2_ = 32-1-1 = 31), according to Fisher's F-distribution table, equals 1.89. Since F_obs_ < F_tabl_ (1.4452 < 1.89), the F-test has not been passed at the significance level of 0.05.

**Table 6:** Results of regression analysis of dependence z_1_(x).

Regression statistics	
Multiple R	0.2144
R-square	0.0460
Adjusted R-square	0.0142
Standard error	0.0486
Observations	32

Dispersion analysis					
	df	SS	MS	F	Significance F
Regression	1	0.0034	0.0034	1.4452	0.2387
Residue	30	0.0708	0.0024		
Total	31	0.0743			

	Coefficients	Standard error	t-statistics	R-Value	Lower 95%	Upper 95%
Y-intercept	0.9005	0.0102	88.0526	0.0000	0.8796	0.9065
x1	0.0924	0.0769	1.2022	0.2387	-0.0646	0.3074

According to the data of Table 7, venture capital does not have a statistically significant influence on innovative development strategies (regression model y_5_ = F(x), Table 6), as significance F = 0.0116 (exceeds 0.05), the change of dependent variable is by 19.41% explained by the change of independent variable (R^2^ = 0.1941). The estimated coefficient gained a positive value of 30.7468. Fobs equals 7.2264. F_tabl_, with 32 observations (k_1_ = 32) and 1 variable (k_2_ = 32-1-1 = 31), according to Fisher's F-distribution table, equals 1.89. Since F_obs_ > F_tabl_ (7.2264 > 1.89), the F-test has been successfully passed at the significance level of 0.05.

**Table 7:** Results of regression analysis of dependence z_2_(x).

Regression statistics	
Multiple R	0.4406
R-square	0.1941
Adjusted R-square	0.1673
Standard error	7.2299
Observations	32

Dispersion analysis					
	df	SS	MS	F	Significance F
Regression	1	377.7297	377.7297	7.2264	0.0116
Residue	30	1,568.1315	52.2711		
Total	31	1,945.8612			

	Coefficients	Standard error	t-statistics	R-Value	Lower 95%	Upper 95%
Y-intercept	47.7748	1.5216	31.3983	0.0000	44.6674	53.3097
x1	30.7468	11.4377	2.6882	0.0116	7.3878	59.7040

According to the data of Table 8, venture capital has a statistically significant influence on digital competitiveness (regression model y_6_ = F(x), Table 7), as significance F = 0.0027 (exceeds 0.05), and the change of dependent variable is by 26.32% explained by the change of independent variable (R^2^ = 0.2632). The connection between the indicators is direct (estimate coefficient gains positive value 61.3670). Fobs equals 10.7166. F_tabl_, with 32 observations (k_1_ = 32) and 1 variable (k_2_ = 32-1-1 = 31), according to Fisher's F-distribution table, equals 1.89. Since Fobs > Ftabl (10.7166 > 1.89), the F-test has been successfully passed at the significance level of 0.05.

**Table 8:** Results of regression analysis of dependence z_3_(x).

Regression statistics	
Multiple R	0.5130
R-square	0.2632
Adjusted R-square	0.2386
Standard error	11.8494
Observations	32

Dispersion analysis					
	df	SS	MS	F	Significance F
Regression	1	1,504.7009	1,504.7009	10.7166	0.0027
Residue	30	4,212.2629	140.4088		
Total	31	5,716.9638			

	Coefficients	Standard error	t-statistics	R-Value	Lower 95%	Upper 95%
Y-intercept	73.1634	2.4938	29.3382	0.0000	68.0704	83.3998
x1	61.3670	18.7459	3.2736	0.0027	23.0827	109.8590

**Figure 1:**
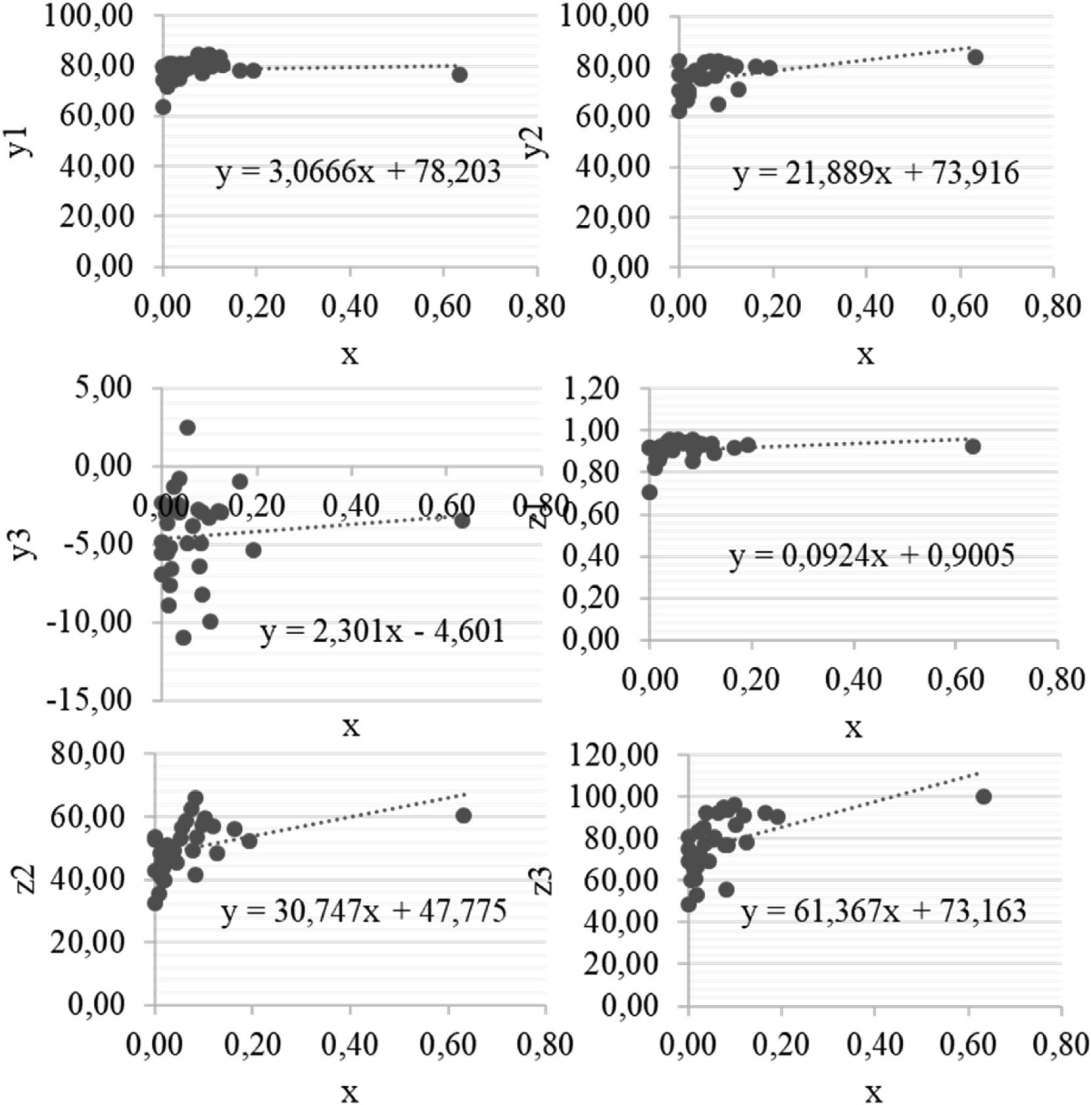
Regression curves that reflect dependence y_1_-y_6_ on x. Source: Authors.

Standard errors in White's form in all regression models (according to Tables 3, 4, 5, 6, 7, and 8) are moderate and amounted to 6.2416, 8.6948, 4.52, 0.0769, 11.4377 and 18.7459, respectively, which confirms the reliability of the models. Hence, econometric models are consistent with the Gauss-Markov theorem. We built regression curves to present the findings, which reflect the dependence y_1_-y_6_ on x (Figure 1).

The results from Tables 3, 4, 5, 6, 7, and 8 and Figure 1 confirm the Hypothesis: venture capital has a substantial impact on economic and public well-being growth, slightly stimulating the achievement of this growth's specific goals. First, support for implementing the SDGs. Second, the formation of competitive advantages due to automatisation and precision of production and consumption. Third, venture capital may stimulate economic growth (accelerated growth of GDP). Fourth is the development of labour potential and the most effective use of human capital. Thus, the role of education and creative capabilities grows, and companies create conditions for their usage. Fifth, venture capital can stimulate innovations: the knowledge society and knowledge economy (innovative economy).

Sixth, venture capital can strengthen digital competitive advantages and positions in the high-tech world markets. While the directions of usage of venture capital are differentiated, and the number of implemented projects in the digital sphere is low, in the future, most innovative projects in Industry 4.0 will envisage the implementation of breakthrough digital technologies.

## 5 Discussion

The above conclusions and recommendations develop the investment theory, specifying venture capital's current and future contribution to economic and public well-being growth (Table 9).

**Table 9:** Transformation of the role of venture capital in the achievement of the SDGs.

SDGs	Role of venture capital in the achievement of the SDGs	
	Before the Fourth industrial revolution (2019)	After the Fourth industrial revolution (Industry 4.0)
Achieving and supporting SGDs	minimum: financing of specific projects in “green” innovations	high: increase of energy and resource efficiency of companies that transferred to Industry 4.0
Achieving and supporting the global competitiveness of the economy	moderate: financing of unique national projects	high: most of the financed projects stimulate the increase of global competitiveness
Accelerating the rate of economic growth	minimum: financial support for specific subjects of small and medium entrepreneurship	high: financing of Industry 4.0 as a determinant of economic growth
Development and implementation of human potential	minimum: funding for the development of human resources of individual companies	high: most of the financed projects lead to the creation of highly efficient jobs
Innovative development of the economy	minimum: financial support for specific creative projects	high: most of the funded projects belong to hi-tech
Supporting and increasing digital competitiveness of the economy	moderate: financing of the most perspective projects in the digital sector	high: most of the financed projects belong to the digital sector

As shown in Table 9, after the Fourth Industrial Revolution, the contribution of venture capital to the achievement of the SDGs becomes high due to integrating the Sustainable Development Goals in placement and venture capital in Industry 4.0. The results emphasize the considerable potential of maximising the contribution of Industry 4.0 to sustainable development. However, the regression analysis results have shown that this potential has not been fully implemented. Thus, there is a need for more active government interference with the Fourth industrial revolution to integrate the Sustainable Development Goals in Industry 4.0 through venture capital regulation.

## 6 Limitations and future research

The research limitations are connected to the fact that Industry 4.0 venture capital will not help achieve this growth. We cannot reach sustainable development, economic growth, and implementing human goals. However, the following goals could be achieved: innovative development, global competitiveness, and increasing digital competitiveness.

Another limitation is that we obtained research findings from a sample of developed countries; therefore, our conclusions can partially be applied to developing countries.

Future research perspectives comprise the search for alternative sources of achieving the goals of growth of Industry 4.0 that cannot be reached based on venture capital, notably sustainable development, economic growth, and implementation of human potential. Also, further research should study the experience of the influence of Industry 4.0 venture capital of developing countries’ economies.

## 7 Conclusions

Our paper conjectured that Industry 4.0 venture capital would help achieve all growth goals: sustainable and innovative development, global competitiveness, implementation of human potential, and increased digital competitiveness. Overall, we found that venture capital would get a creative role and a "wide" treatment, which goes beyond the limits of the economy. Besides the traditionally acknowledged economic advantages – i.e., the formation of competitive advantages and the strengthening of digital competitive advantages and positions in the high-tech world markets – venture capital also benefits economic growth. It generates non-economic (social) advantages by supporting the accomplishment of the SDGs, development of labour potential, the most effective use of human capital, and the development of the knowledge society and knowledge economy.

This paper's proposed new – “wide” – treatment of venture capital 4.0 allows improving venture investors' market strategies and strives to provide a whole range of advantages for the interested parties. Due to this, venture capital 4.0 will become a tool of corporate social responsibility. This opens a new scope for future research and emphasizes the theoretical significance of this paper.

The importance of policymaking is to show the critical importance of the Fourth Industrial Revolution's financial provision (based on venture capital) to benefit from it, particularly as it increases global and digital competitiveness. The empirical data and the performed econometric analysis confirm the existing volume of the corresponding knowledge, proving that the venture capital of Industry 4.0 contributes to social and economic development. The article's results supplement and develop the current understanding and contribute to the literature, demonstrating the systemic influence of Industry 4.0 venture capital, ensuring the integration of the Sustainable Development Goals in Industry 4.0 and the extreme relevant balance between environment, economy, and society.

## Declarations

### Author contribution statement

Elena G. Popkova: Conceived and designed the experiments; Performed the experiments; Wrote the paper.

Bruno S. Sergi: Analyzed and interpreted the data; Wrote the paper.

### Funding statement

The authors received no funding from an external source.

### Data availability statement

Data will be made available on request.

### Declaration of interests statement

The authors declare no conflict of interest.

### Additional information

No additional information is available for this paper.
